# Preliminary Evidence for the Cognitive Model of Auditory Verbal Hallucinations in Youth With Borderline Personality Disorder

**DOI:** 10.3389/fpsyt.2019.00292

**Published:** 2019-05-16

**Authors:** Marialuisa Cavelti, Katherine Thompson, Carol Hulbert, Jennifer Betts, Henry Jackson, Shona Francey, Andrew Chanen

**Affiliations:** ^1^Orygen, The National Centre of Excellence in Youth Mental Health, Melbourne, VIC, Australia; ^2^Centre for Youth Mental Health, The University of Melbourne, Melbourne, VIC, Australia; ^3^Melbourne School of Psychological Sciences, The University of Melbourne, Melbourne, VIC, Australia; ^4^University Hospital for Child and Adolescent Psychiatry and Psychotherapy, The University of Bern, Bern, Switzerland; ^5^Orygen Youth Health, Melbourne, VIC, Australia

**Keywords:** borderline personality disorder, schizophrenia, psychosis, auditory hallucinations, beliefs about voices, distress, depression, anxiety

## Abstract

**Objectives:** This is the first study to explore cognitive, emotional, and behavioral responses to voices in youth with borderline personality disorder (BPD) compared with those with schizophrenia spectrum disorder (SZ), and to examine if negative appraisals of voices predict depression and anxiety across the groups.

**Methods:** The sample comprised 43 outpatients, aged 15–25 years, who reported auditory verbal hallucinations (AVH) and were diagnosed with either *Diagnostic and Statistical Manual of Mental Disorders, 5th Edition* (DSM-5) BPD or SZ. Data were collected using the Psychotic Symptom Rating Scales, the revised Beliefs About Voices Questionnaire, the Voice Rank Scale, and the Depression Anxiety Stress Scale.

**Results:** Youth with BPD did not differ from youth with SZ in beliefs about the benevolence or malevolence of voices. Youth with BPD appraised their voices as more omnipotent and of higher social rank in relation to themselves, compared with youth with SZ. In both diagnostic groups, beliefs about malevolence and omnipotence of voices were correlated with more resistance toward voices, and beliefs about benevolence with more engagement with voices. In addition, perceiving the voices as being of higher social rank than oneself and negative voice content were both independent predictors of depression, irrespective of diagnostic group. In contrast, negative appraisals of voices did not predict anxiety after adjusting for negative voice content.

**Conclusions:** This study replicated the link between negative appraisals of voices and depression that has been found in adults with SZ in a mixed diagnostic youth sample. It, thus, provides preliminary evidence that the cognitive model of AVH can be applied to understanding and treating voices in youth with BPD.

## Introduction

Increasing evidence suggests that auditory verbal hallucinations (AVH) are common and highly distressing in adults with borderline personality disorder (BPD) (1–6). Although the cognitive model of AVH (7, 8) has informed the development of psychological treatments, such as cognitive behavioral therapy (CBT) for patients with schizophrenia, few studies have examined the usefulness of this model for the understanding and treatment of voices in BPD. None have done this in young patients early in the course of BPD. This study aimed to explore the cognitive model of AVH in youth (aged 15–25 years) with BPD. This age group coincides with the peak period of clinical onset for both BPD (9) and psychotic disorders (10).

Auditory hallucinations have been defined as “auditory experiences that occur in the absence of a corresponding external stimulation and which resemble a veridical perception” (11). If the auditory experiences involve the perception of spoken language, they are referred to as AVH or voices, which is their most common form (12). While AVH are most common in patients with psychotic disorders (40%–80%), there is increasing evidence that they also occur in healthy individuals (10%–20%) and in patients with nonpsychotic mental disorders, including BPD (20%–50%) (3, 11, 13, 14).

Not all individuals reporting AVH seem to be perturbed or impaired by these symptoms. Therefore, the determinants of distress and dysfunction associated with AVH need to be elucidated. Studies comparing AVH in clinical and nonclinical samples have revealed two clear, differentiating factors: voice content and voice appraisal. Patients (i.e., people who seek help for their distressing voices, irrespective of diagnosis) more often report negative voice content (e.g., negative comments, verbal abuse, personal insults, commands to harm oneself or others) and negative appraisals of voices (e.g., as malevolent, powerful, dominant, intrusive, controllable). Consequently, they are more likely to engage in maladaptive coping strategies (e.g., safety behaviors, compliance, resistance, ignorance, distance) than nonpatients (12, 15, 16). This suggests that factors other than the mere presence of the symptom lead to distress (e.g., any negative affect, such as depression, anxiety, or voice-related distress), dysfunction, and need for care. This is consistent with a “continuum hypothesis” of AVH, suggesting that voice-hearing occurs in the general population, as well as in clinical samples, with the latter group reporting higher levels of distress and need for care (15). Studies examining the differences between the two groups found that it is not the presence of AVH *per se*, but rather the negative voice content and the negative appraisals of voices that determine the level of distress and need for care [e.g., Ref. (16)].

Chadwick and Birchwood (7, 8) observed that, in patients with schizophrenia, beliefs about voices often involve the person making inferences beyond what is manifest in voice content alone. Consequently, the cognitive model of AVH asserts that the way an individual cognitively appraises their voices is the primary determinant of emotional and behavioral responses to the experience (7, 8). In support of this, *cognitive appraisals* of voices in terms of malevolent intention, power, and social rank have been associated with more resistance to (in contrast to engagement with) and higher levels of voice-related distress, anxiety, and depression among voice-hearers with schizophrenia and related disorders, irrespective of *form* (e.g., frequency, duration, location, loudness) or *content* of voices (17–23). Mawson et al. (24) reviewed the literature regarding the cognitive model of AVH and concluded that the relationship between appraisals of malevolence (i.e., intent of voices to harm) and supremacy of voices (i.e., omnipotence, social power, and rank of voices compared to voice-hearer) with distress received the most empirical support. The clinical implication is that making *cognitive appraisals* of voices the target of psychological interventions, rather than the *form* or *content* of voices, might assist reduction of distress and problematic coping behaviors in individuals with AVH.

Recent evidence indicates that AVH in adults with BPD are phenomenologically similar to those in schizophrenia, elicit high levels of distress, depression, and anxiety, and are associated with more psychiatric comorbidity, suicidal plans and attempts, and hospitalizations (2–3, 4, 6, 25–27). Limited evidence exists regarding whether the cognitive, emotional, and behavioral responses to voices in patients with BPD are similar or different to those in patients with schizophrenia. Hepworth et al. (28) reported that adults with BPD did not differ from those with schizophrenia in beliefs about malevolence and omnipotence of voices, or in behavioral resistance and engagement, but showed more emotional resistance toward and less emotional engagement with voices. In another study of adults with BPD, beliefs about malevolence and social rank of voices were correlated with distress, and beliefs about omnipotence of voices were also correlated with distress, along with the number of hospitalizations within 2 years postbaseline, and the number of days until hospitalization (29). These two studies explored cognitive appraisals of voices in adults with longstanding BPD [mean age was 33.70 years (28) and 39 years (29), respectively]. To date, no attention has been given to young people, even though adolescence and early adulthood represent a sensitive period for the development, detection, and early treatment of symptoms associated with both BPD and psychotic disorders, such as AVH (9, 10). Information about the cognitive, emotional, and behavioral responses to voices across the lifespan might inform clinical practice regarding whether a transdiagnostic, symptom-focused treatment approach is appropriate.

In a recent study, our group explored AVH in a sample of outpatient youth (15 to 25 years of age) with BPD and found that they were similar to those in youth with schizophrenia spectrum disorder (SZ) with regard to physical (frequency, duration, location, loudness), cognitive (beliefs regarding origin of voices, disruption to life, controllability), and emotional (negative content, distress) characteristics (30). Using this same sample, the current study aimed to investigate whether beliefs, emotions, and behaviors associated with AVH in youth with BPD are similar to or different from those in youth with schizophrenia spectrum disorder (exploratory aim 1). Based on the literature in adult patients, we hypothesized that youth with BPD will show higher levels of emotional resistance toward voices, depression, and anxiety compared to youth with schizophrenia spectrum disorder (Hypothesis 1). We also examined whether the assumptions of the cognitive model of AVH might apply, regardless of diagnostic group (BPD or SZ) (exploratory aim 2). Based on the literature in adult patients, the following hypotheses were tested: Beliefs about malevolence and omnipotence of voices will be related to resistance toward voices, while beliefs about benevolence of voices will be related to engagement with voices, irrespective of diagnosis (Hypothesis 2). Negative appraisals of voices (in terms of malevolence, omnipotence, and high social rank) will predict high levels of depression and anxiety, after adjusting for the impact of form and content of voices, irrespective of diagnosis (Hypothesis 3).

## Materials and Methods

### Participants

Forty-three help-seeking youth, aged 15–25 years, with AVH, who were diagnosed with either BPD (BPD+AVH; *n* = 23) or schizophrenia spectrum disorder (SZ) (SZ+AVH, *n* = 20) according to the *Diagnostic and Statistical Manual of Mental Disorders*, *5th Edition* (DSM-5) (31) and were fluent in English, participated in the study. They constituted a subsample of a study that has been reported elsewhere (30). Two participants from the original SZ+AVH group (*n* = 22) did not complete the questionnaires, and were thus excluded from these analyses. AVH were defined as present according to the threshold set in the Comprehensive Assessment of At Risk Mental States (CAARMS) (32) for more than 1 week within the past 3 months. Threshold AVH are defined in the CAARMS as an intensity rating of 5 or higher and a frequency rating of 4 or higher on the Perceptual Abnormalities subscale. This corresponds to hearing voices i) at least three times a week for more than an hour per occasion; or ii) daily for any duration per occasion.

The BPD+AVH group included youth with a DSM-5 BPD diagnosis and CAARMS threshold AVH. Participants were excluded from this group if they were diagnosed with a DSM-5 delusional disorder, schizophreniform disorder, schizophrenia, schizoaffective disorder, substance/medication-induced psychotic disorder, psychotic disorder due to another medical condition, catatonia, or bipolar I disorder.

The SZ+AVH group included youth with a DSM-5 brief psychotic disorder, schizophreniform disorder, schizophrenia, or schizoaffective disorder and CAARMS threshold AVH. Exclusion criteria for this group were a DSM-5 delusional disorder, substance/medication-induced psychotic disorder, psychotic disorder due to another medical condition, catatonia, or bipolar I disorder, or having more than two DSM-5 BPD criteria.

### Procedure

Participants were recruited between June 2016 and February 2018 from Orygen Youth Health, the state government-funded specialist mental health service for 15–25 year olds living in northwestern and western metropolitan Melbourne, Australia. The service includes specialist early intervention programs for psychosis (33) and for BPD (34). In accordance with the Declaration of Helsinki, written informed consent was obtained from all participants, and additionally from a parent or guardian for those under 18 years old. Participants were interviewed by a clinical psychologist-researcher or by graduate research assistants who were specifically trained in the application of the measures. Participants were reimbursed for time and expenses. The study was approved by the Melbourne Health Human Research Ethics Committee (MHREC2016.086).

### Measures

Participants were assessed using the positive symptom scales of the CAARMS, a semistructured interview conducted to determine the presence, type, frequency, and severity of subthreshold and threshold psychotic symptoms (32). The Perceptual Abnormalities subscale was used to assess AVH as described above. The modules A–D (affective and psychotic disorders) of the Structured Clinical Interview for DSM-5, Research Version (SCID-5-RV) (35) and the BPD section of the Structured Clinical Interview for DSM-5 Personality Disorders (SCID-5-PD) (36) were administered to establish diagnostic status.

After establishing eligibility for the study, a series of interviews and questionnaires, as described below, were administered and demographic data were collected. Residential postcode was used to determine socioeconomic status according to an Australian index of socioeconomic disadvantage (37). The tertiles of the rank (i.e., low, middle, and high socioeconomic status) were used for analyses.

General psychosocial functioning was assessed using the Social and Occupational Functioning Assessment Scale (SOFAS) (38), which ranges from 1 (persistent instability to maintain minimal personal hygiene, unable to function without harming self or others or without considerable external support) to 100 (superior functioning in a wide range of activities).

Phenomenological characteristics of AVH were assessed using the Auditory Hallucinations subscale of the Psychotic Symptom Rating Scales (PSYRATS-AH) (39). It consists of 11 items, rated on a five-point scale (0–4). The items assessing form (i.e., frequency, duration, location, and loudness) and content (i.e., amount of negative voice content, and degree of negative voice content) were used for the current analyses.

The 21-item version of the Depression Anxiety Stress Scale (DASS-21) (40) was administered to assess distress over the past week. For the current analyses, only the depression and anxiety subscales were used. The depression subscale measures symptoms typically associated with dysphoric mood (e.g., sadness or worthlessness), while the anxiety subscale measures symptoms of physical arousal, panic attacks, and fear (e.g., trembling or faintness). The items are rated on a four-point Likert scale (0 = did not apply to me at all, to 3 = applied to me very much, or most of the time). The Cronbach’s alpha scores in the current study were 0.94 and 0.87 for depression and anxiety, respectively, indicating excellent internal consistency. The depression and anxiety subscales of the DASS-21 were used as outcome variables in this study measuring amount and intensity of distress instead of the PSYRATS-AH items because a) the DASS-21 subscales are continuous in contrast to the four-point Likert scale of the PSYRATS items, and b) previous research found that nearly two-thirds of voice-hearers diagnosed with schizophrenia experience at least moderate depression (17) and that AVH is associated with increased levels of depression and anxiety in adults with BPD, too (27).

Cognitive, emotional, and behavioral responses to voices were explored using the revised Beliefs About Voices Questionnaire (BAVQ-R) (18). It consists of 35 items rated on a four-point Likert scale (0 = disagree to 3 = strongly agree). There are five subscales, three relating to beliefs about voices (i.e., malevolence, benevolence, and omnipotence) and two relating to emotional and behavior responses to voices (i.e., engagement and resistance). The beliefs subscales each consists of six items. The resistance subscale includes five items on emotion and four on behavior, while the engagement subscale includes four items on emotion and four on behavior. Cronbach’s alpha scores for the subscales in the current study ranged between 0.72 and 0.89, indicating adequate internal consistency.

The Voice Rank Scale (VRS) (18, 41) uses a semantic differential adapted from the Social Comparison Scale to measure the individual’s rank relative to the dominant voice. The scale consists of 11 items with scores ranging from 1 to 10 (e.g., Incompetent 1 2 3….8 9 10 Component). A low sum score indicates that the individual experiences him-/herself as of lower social rank compared to the voice. Internal consistency of the scale in the current study was good, with Cronbach’s alpha = 0.87.

### Statistical Analyses

Statistical analyses were performed using IBM Statistical package for the social sciences (SPSS) Statistics for Windows, version 22.0 (42). Missing value analyses revealed one missing value in the DASS-21 and the VRS each, as well as three missing values in the BAVQ-R. These missing values were completely at random as indicated by nonsignificant Little’s Missing completely at random (MCAR) tests, and were replaced by expectation maximization methods (43).

Demographic characteristics were compared between the two groups using chi-square tests (education status, employment status), Fisher’s exact tests if expected cell counts of categorical variables were less than five (gender, relationship status, main financial support, socioeconomic status), Mann–Whitney *U* test (SOFAS), and *t*-test for independent samples (age).

In order to examine whether beliefs, emotions, and behaviors associated with AVH in youth with BPD differed from those in youth with SZ and AVH (exploratory aim 1 and Hypothesis 1), group comparisons were performed using the Mann–Whitney *U* test for the BAVQ-R subscales and the DASS-21 subscales, as well as the *t*-test for independent samples for the VRS. Group comparisons of the PSYRATS-AH items have been reported elsewhere (30).

In order to examine whether the assumptions of the cognitive model of AVH apply, regardless of BPD or SZ diagnosis (exploratory aim 2), correlation and regression analyses were conducted. In order to test Hypothesis 2, Spearman’s correlations between the BAVQ-R subscales were conducted on the whole sample. The correlational analyses were then repeated for the BPD+AVH group and the SZ+AVH group separately, and correlation coefficients between the groups were compared using Fisher’s *Z* test adapted for Spearman’s rho in accordance to Sheskin (44).

In order to test Hypothesis 3, Spearman’s correlations were first conducted between potential confounders (gender, age), the PSYRATS-AH items (frequency, duration, loudness, location, amount of negative voice content, degree of negative voice content), the BAVQ-R beliefs about voices subscales (malevolence, benevolence, omnipotence), the VRS, and the DASS-21 depression and anxiety subscales on the whole sample. The correlation analyses were then repeated for the BPD+AVH group and the SZ+AVH group separately, and correlation coefficients between the groups were compared using Fisher’s *Z* test for Spearman’s rho (44). Those variables that were identified as holding a significant correlation with depression or anxiety were used as predictor variables in the subsequent regression analyses. Two hierarchical linear regression analyses were then conducted for depression and anxiety separately. In each analysis, the demographic variables and the PSYRATS-AH items were entered as predictor variables in the first step, and the BAVQ-R subscales and VRS in the second step. Lastly, we conducted a moderation analysis to test if group (BPD+AVH, SZ+AVH) moderated the effects of cognitive appraisals of voices on distress, using SPSS PROCESS macro version 3.00 (45). PROCESS uses ordinary least squares regression to estimate the regression coefficients, and bootstrapping methods for the confidence intervals, yielding results that are less affected by sample size. For each regression analysis, the assumptions of linearity and multicollinearity, as well as of independence, normality, and homoscedasticity of residuals, were checked.

Nonparametric tests were used if variables were not normally distributed across groups, normality could not be achieved through transformation, and/or outliers were detected by visual inspection of box plots. To provide an estimate of the size of observed effects that is independent of sample size and measure used (46), effect sizes (*d*, θ, r, *R* *^2^*, and rs^2^) were computed. θ = U/mn is the generalized Mann–Whitney effect size measure that ranges from 0 to 1, taking the value 0.5 on the null hypothesis (identically distributed) and 0 or 1 if there is no overlap between the two samples (47). Newcombe (48) provided an Excel spreadsheet, which was used to calculate θ and its confidence intervals. Theta values in the range 0.4–0.6 were considered as small, in the ranges 0.61–0.8 and 0.2–0.39 as moderate, and in the ranges 0.81–1 and 0–1.9 as large. The sizes of *d* and r were interpreted according to Cohen (49).

## Results

### Participant Characteristics

The demographic characteristics of participants are presented in **Table 1**. Participants in the BPD+AVH group did not significantly differ from participants in the SZ+AVH group with regard to demographic characteristics, except that participants of the former group were significantly more often female, younger, and enrolled in education.

**Table 1:** Participant demographics.

	BPD+AVH (*n* = 23)	SZ+AVH (*n* = 20)	Group differences	
	M (SD)/*n*(%)	M (SD)/*n*(%)	Test statistic	*p*
Gender				.001**
Male Female	1 (4.3) 22 (95.7)	10 (50.0) 10 (50.0)		
Age (years)	18.13 (2.30)	20.00 (3.15)	*t*(41) = 2.24	.030*
Romantic relationship	8 (34.8)	4 (20.0)		.327
In education	17 (73.9)	8 (40.0)	X^2^(1) = 5.06	.033*
Employed	8 (34.8)	7 (35.0)	X^2^(1) = 0.00	1.00
Main financial support			X^2^ = 0.45	.853
Employment Acquaintances Government benefits	4 (17.4) 10 (43.5) 9 (39.1)	5 (25.0) 8 (40.0) 7 (35.0)		
Socioeconomic status			X^2^ = 1.86	.395
Low Middle High	10 (43.5) 9 (39.1) 4 (17.4)	5 (25.0) 9 (45.0) 6 (30.0)		
Psychosocial functioning	52.74 (12.16)	54.30 (8.52)	*U* = 223.00	.864

AVH, auditory verbal hallucinations; BPD, borderline personality disorder; SOFAS, Social and Occupational Functioning Assessment Scale; SZ, schizophrenia spectrum disorder. Significant at: *p < .05; **p < .01.

SZ+AVH group participants were diagnosed with the following psychotic disorders: 1 (5.0%) with brief psychotic disorder, 5 (25.0%) with schizophreniform disorder, 3 (15.0%) with schizoaffective disorder, and 11 (55.0%) with schizophrenia. A comprehensive characterization of the groups in terms of psychotic symptoms is reported elsewhere (30). In short, AVH, as assessed by the PSYRATS-AH items, in the BPD+AVH group were found to be phenomenologically indistinguishable from those in the SZ+AVH group (see **Table S1**).

### Cognitive, Emotional, and Behavioral Responses to Voices and Depression and Anxiety in Youth With Borderline Personality Disorder Compared With Those With Schizophrenia Spectrum Disorder (Exploratory Aim 1, Hypothesis 1)

**Table 2** presents the results of the group comparisons of the BAVQ-R subscales, the VRS, and the DASS-21 depression and anxiety subscales. Participants in the BPD+AVH group significantly more often appraised their voices as omnipotent, of higher social rank than themselves, and reported more symptoms of depression and anxiety, than did participants in the SZ+AVH group. The effect sizes for these group differences were medium to large. There were no statistically significant group differences in beliefs about malevolence and benevolence of voices, or in emotional or behavioral responses to voices (i.e., resistance, engagement), and these effect sizes were small to medium.

**Table 2:** Group differences in cognitive, emotional, and behavioral responses to voices, depression, and anxiety.

	BPD+AVH (*n* = 23)			SZ+AVH (*n* = 20)			Group differences		
	M (SD)	Mnd	MR	M (SD)	Mnd	MR	Test statistic	*p*	ES and (95%) CI
BAVQ-R Malevolence	11.06 (5.24)	11.00	24.87	8.90 (4.42)	8.00	18.92	*U* = 291.50	.133	θ = 0.63 (0.46, 0.78)
BAVQ-R Benevolence	4.26 (4.85)	2.00	21.67	4.55 (4.83)	3.00	22.38	*U* = 222.50	.852	θ = 0.48 (0.32, 0.65)
BAVQ-R Omnipotence	12.85 (4.11)	13.00	26.15	10.35 (3.42)	11.00	17.22	*U* = 325.50	.019*	θ = 0.71 (0.53, 0.83)
BAVQ-R Emotional resistance	9.11 (2.75)	9.00	24.85	7.90 (2.97)	8.00	18.95	*U* = 291.00	.133	θ = 0.63 (0.46, 0.77)
BAVQ-R Behavioral resistance	9.83 (4.10)	10.00	22.07	10.05 (2.70)	10.50	21.92	*U* = 231.50	.971	θ = 0.50 (0.34, 0.67)
BAVQ-R Emotional engagement	2.35 (3.11)	1.00	20.24	3.40 (3.50)	3.00	24.02	*U* = 189.50	.308	θ = 0.41 (0.26, 0.58)
BAVQ-R Behavioral engagement	2.30 (2.98)	1.00	20.46	3.20 (3.41)	2.00	23.78	*U* = 194.50	.377	θ = 0.42 (0.27, 0.59)
Voice Rank Scale	36.50 (15.03)	N/A	N/A	49.45 (15.92)	N/A	N/A	*t*(41) = 2.74	.009**	*d* = 0.84 (0.21, 1.46)
DASS-21 Depression	15.26 (4.97)	16.00	28.48	7.60 (6.44)	6.50	14.55	*U* = 379.00	.000***	θ = 0.82 (0.66, 0.91)
DASS-21 Anxiety	13.74 (4.84)	13.00	29.24	6.70 (4.24)	6.50	13.68	*U* = 396.50	.000***	θ = 0.86 (0.71, 0.94)

BAVQ-R, revised Beliefs About Voices Questionnaire; DASS-21, Depression Anxiety Stress Scales; N/A, not applicable. Significant at: *p < .05; **p < .01; ***p < .001.

### Relationship Between Negative Appraisals of Voices and Emotional and Behavioral Responses to Voices in Youth With Borderline Personality Disorder Compared With Those With Schizophrenia Spectrum Disorder (Exploratory Aim 2, Hypothesis 2)

**Table 3** shows the correlations between the BAVQ-R subscales assessing beliefs about voices and the subscales assessing emotional and behavioral responses to voices for the whole sample. Malevolence and omnipotence were moderately to strongly correlated with more emotional resistance. In addition, malevolence was moderately correlated with less emotional engagement, and omnipotence with more behavioral resistance. In contrast, benevolence was moderately correlated with less emotional resistance, and strongly correlated with more emotional and behavioral engagement.

**Table 3:** Relationships between beliefs about voices and emotional and behavioral responses to them (*n* = 43).

	BAVQ-R Emotional resistance			BAVQ-R Behavioral resistance			BAVQ-R Emotional engagement			BAVQ-R Behavioral engagement		
	r_s_	*p*	95% CI	r_s_	*p*	95% CI	r_s_	*p*	95% CI	r_s_	*p*	95% CI
BAVQ-R Malevolence	.61	.000***	0.38, 0.77	.26	.092	−0.04, 0.52	−.36	.019*	−0.60, −0.07	−.25	.106	−0.51, 0.05
BAVQ-R Benevolence	−.40	.008**	−0.63, −0.11	−.19	.228	−0.46, 0.12	.79	.000***	0.64, 0.88	.72	.000***	0.54, 0.84
BAVQ-R Omnipotence	.45	.003**	0.17, 0.66	.44	.003**	0.16, 0.65	−.19	.216	−0.46, 0.12	−.09	.561	−0.38, 0.22

BAVQ-R, revised Beliefs About Voices Questionnaire. Significant at: *p < .05; **p < .01; ***p < .001.

A comparison of correlations between the BPD+AVH group and the SZ+AVH group revealed a significant group difference in the correlation between malevolence and emotional engagement (*p* = .014) only. The relationship between these two variables was large and significant in the SZ+AVH group (r_s_ = −.71, *p* < .000, 95% CI [−0.88, −0.39]), and negligible and not significant in the BPD+AVH group (r_s_ = −.04, *p* = .843, 95% CI [−0.47, 0.41]).

### Relationship Between Negative Appraisals of Voices and Depression and Anxiety in Youth With Borderline Personality Disorder Compared to Those With Schizophrenia Spectrum Disorder (Exploratory Aim 2, Hypothesis 3)

As seen in **Table 4**, depression was moderately to strongly correlated with being female, a higher amount and degree of negative voice content, and more negative appraisals of voices in terms of malevolence, omnipotence, and social rank. Anxiety was moderately correlated with the degree of negative voice content and negative appraisals of voices (malevolence, omnipotence, voice social rank). The comparison of the correlations between the BPD+AVH group and the SZ+AVH group revealed no significant differences (*p* > .05).

**Table 4:** Relationship between demographic variables, voice form and content, and cognitive appraisals of voices with depression and anxiety (*n* = 43).

	DASS-21 Depression			DASS-21 Anxiety		
	r_s_	*p*	95% CI	r_s_	*p*	95% CI
Gender	.36	.018*	0.07, 0.60	.29	.061	−0.01, 0.54
Age	−.23	.140	−0.50, 0.08	−.09	.548	−0.38, 0.22
PSYRATS-AH frequency	.07	.662	−0.24, 0.36	−.12	.462	−0.41, 0.19
PSYRATS-AH duration	.28	.070	−0.02, 0.54	.05	.752	−0.25, 0.35
PSYRATS-AH location	−.07	.653	−0.36, 0.24	−.15	.325	−0.43, 0.16
PSYRATS-AH loudness	.20	.205	−0.11, 0.47	.23	.141	−0.08, 0.49
PSYRATS-AH amount of negative voice content	.37	.014*	0.08, 0.60	.23	.143	−0.08, 0.49
PSYRATS-AH degree of negative voice content	.66	.000***	0.45, 0.80	.34	.025*	0.05, 0.58
BAVQ-R Malevolence	.52	.000***	0.26, 0.71	.35	.021*	0.06, 0.59
BAVQ-R Benevolence	−.22	.159	−0.49, 0.09	.01	.97	−0.29, 0.31
BAVQ-R Omnipotence	.41	.007**	0.13, 0.63	.40	.008**	0.11, 0.63
VRS	−.49	.001**	−0.69, −0.22	−.34	.025*	−0.58, −0.05

BAVQ-R, revised Beliefs About Voices Questionnaire; DASS, Depression Anxiety Stress Scale; PSYRATS-AH, Psychotic Symptom Rating Scales Auditory Hallucinations; VRS, Voice Rank Scale. Significant at: *p < .05; **p < .01; ***p < .001.

The results of the hierarchical regression analyses examining whether the addition of negative appraisals of voices improved the prediction of depression and anxiety, over and above gender and/or voice content, are summarized in **Table 5**. The estimated proportion of variance explained by gender and/or negative voice content alone was 40% for depression and 11% for anxiety. Entering negative appraisals of voices in the second step explained significant additional variance for depression only. The estimated proportion of variance explained by negative appraisals of voices was 19% for depression and 7% for anxiety. In the final model, depression was significantly predicted by the degree of negative voice content and perceived social rank of voices, which explained 16% and 11% of variance, respectively.

**Table 5:** Hierarchical regression analyses predicting depression and anxiety in youth with AVH who were either diagnosed with BPD or SZ (*n* = 43).

	B	*β*	*t*	*rs^2^*	*R_a_^2^*	95% CI	*F*	*ΔR^2^*	*ΔF*
DASS-21 Depression									
Step 1 Sex PSYRATS-AH Amount of negative voice content PSYRATS-AH Degree of negative voice content	3.64 −0.37 4.34	.23 −.06 .62	1.92 −0.38 3.75**	.05 .00 .20	.40	0.13, 0.59	10.45***	.45	10.45***
Step 2 Sex PSYRATS-AH Amount of negative voice content PSYRATS-AH Degree of negative voice content BAVQ-R Malevolence BAVQ-R Omnipotence VRS	2.36 −1.16 4.13.20 18.31 −0.04 −0.16	.15 −.20 .59 .23 −.02 −.38	1.39 −1.35 0.41*** 1.65 −0.15 −3.36**	.02 .02 .16 .03 .00 .11	.59	0.32, 0.74	11.18***	.21	7.05**
DASS-21 Anxiety									
Step 1 PSYRATS-AH Degree of negative voice content	2.14	.36	2.51*	.13	.11	0.0, 0.33	6.27*	.13	6.27*
Step 2 PSYRATS-AH Degree of negative voice content BAVQ-R Malevolence BAVQ-R Omnipotence VRS	1.48 0.10 0.25 −0.08	.25 .08 .17 −.24	1.69 0.46 0.97 −1.59	.06 .00 .02 .05	.18	0.0, 0.38	3.32*	.13	2.16

BAVQ-R, revised Beliefs About Voices Questionnaire; DASS, Depression Anxiety Stress Scale; PSYRATS-AH, Psychotic Symptom Rating Scales Auditory Hallucinations; VRS, Voice Rank Scale. Significant at: *p < .05; **p < .01; ***p < .001.

Finally, three moderation analyses were conducted first for depression as the dependent variable and then repeated for anxiety as the dependent variable, in order to examine if the effect of malevolence, omnipotence, or perceived social rank of voices on depression or anxiety differed according to diagnostic group (BPD+AVH versus SZ+AVH). None of the interaction effects of malevolence (ဃ*β* = −.49, *p* = .097, 95% CI [−1.08, 0.09]), omnipotence (ဃ*β* = −.47, *p* = .219, 95% CI [−1.24, 0.29]), or perceived social rank of voices (င*β* = .17, *p* = .110, 95% CI [−0.04, 0.38]) with group on depression was significant. Similarly, no significant interaction effects for malevolence (ဃ*β* = −.11, *p* = .737, 95% CI [−0.78, 0.56]), omnipotence (ဃ*β* = −.44, *p* = .278, 95% CI [−0.37, 1.24]), or perceived social rank of voices (ဃ*β* = .05, *p* = .638, 95% CI [−0.17, 0.27]) with group on anxiety was found. These results indicate that the associations between negative appraisals of voices and depression or anxiety did not differ according to diagnostic group.

## Discussion

This study tested the cognitive model of AVH (7, 8) in youth voice-hearers with BPD or SZ. Overall, the results indicate that the cognitive model of AVH is applicable to the understanding and treatment of voices in youth, regardless of BPD or SZ diagnosis.

Concerning the first exploratory aim, this study found that youth with BPD showed similar beliefs about the benevolence or malevolence of voices, and similar emotional or behavioral responses to voices as youth with SZ. However, youth with BPD appraised their voices as being more omnipotent and of higher social rank than themselves. While the BAVQ-R subscale scores and the Voice Rank Scale scores in the current sample of youth with BPD are broadly comparable to those found in two studies of adults with BPD (28, 29), the current findings also differ in two aspects. First, contrary to the first hypothesis, the finding that adults with BPD and SZ differ in their specific emotional responses to voices, with more emotional resistance and less emotional engagement in the BPD group (28), was not replicated in the current youth sample. Instead, youth with BPD reported higher levels of depression and anxiety than those with SZ. These divergent findings might occur because young voice-hearers do not differ in their initial specific emotional response to voices, and that differences emerge over time as a result of the individual experience of hearing voices (e.g., the individual’s appraisal of voices as malevolent and powerful, and ability to cope with the voices). However, the most likely explanation for the nonsignificant group differences regarding emotional responses to voices in the current study was insufficient statistical power to reliably detect such differences, as both the effect sizes and the sample size were small (50). Indeed, the achieved power to detect a significant group difference with α = .05 was 53% for emotional resistance and 34% for emotional engagement. Second, the finding that appraisals of supremacy of voices (i.e., omnipotence, social rank of voices compared with oneself) were more prominent in youth with BPD than in those with SZ is novel. In patients with SZ, appraisals of supremacy of voices have been found to mirror schema of social power and rank, and together they have been strongly linked to voice-related distress and depression (17, 41). Given that disturbances in the self-concept and interpersonal relationships are key features of BPD (51), it would be interesting to investigate if the appraisals of supremacy of voices found to be prominent among youth with BPD are influenced by negative schema of self and others.

In support of the second hypothesis, the findings show that, in youth with AVH, beliefs about malevolence and omnipotence of voices were correlated with more emotional resistance toward voices, while beliefs about benevolence of voices were associated with more emotional and behavioral engagement with voices. The correlations were similar across diagnostic groups (BPD versus SZ). These findings replicate findings from studies of adults with SZ and AVH, reporting that malevolence and omnipotence were related to resistance, and benevolence to engagement (18, 21–22, 23, 52).

The third hypothesis tested the assumption that it is the way individuals *appraise* their voices, rather than the *form* or *content* of voices, that determines the level of distress experienced by the voice-hearer (7, 8). In partial support of this, frequency, duration, location, and loudness of voices reported in the combined youth sample were weakly (and not significantly) correlated with depression and anxiety, while negative voice content and negative appraisals of voices (i.e., malevolence, omnipotence, high social rank) were moderately to strongly correlated with depression and anxiety. Further, the negative appraisals of voices explained additional variance in depression, over and above the amount explained by negative voice content. However, negative appraisals of voices did not predict anxiety after controlling for negative voice content. Diagnostic group (BPD versus SZ) did not influence the findings. These findings are partially consistent with studies in adults with SZ and AVH reporting that negative appraisals of voices predict both depression and anxiety (18, 22, 23), or depression only (21). A potential explanation for the nonsignificant finding regarding anxiety in the current study is that appraisals of supremacy of voices (i.e., beliefs about power and social rank) render voice-hearers specifically vulnerable for symptoms of depression. Those who perceive their voices as powerful and of higher social rank than themselves might be more likely to experience themselves as powerless, helpless, entrapped, and defeated, and to subordinate themselves to their voices, a state of mind that resembles depression (17, 41). Consistent with this, findings from the current study show that a) negative appraisals of voices were important predictors of depression, but not of anxiety, and b) perceived social rank of voices was a more important predictor of depression than beliefs about malevolence of voices. Finally, the current findings in the combined youth sample replicate the finding that negative voice content influences negative beliefs about voices (23, 53, 54) and voice-related distress (55, 56) in adults with SZ. This suggests that *both voice content and beliefs about voices*—as well as their potential interplay—should be considered as determinants of distress in voice-hearers in research and treatment.

Taken together, the current study provides preliminary evidence that the cognitive model can be applied to the understanding of AVH in youth, regardless of diagnosis of BPD or SZ. This provisional conclusion needs further examination due to the following limitations of the study. First, the sample size was small, which reduced the power of the study to reliably detect group differences (50). For instance, the achieved power to detect incremental changes in *R* ^2^ by adding the interaction terms group×VRS, group×malevolence, and group×omnipotence to the regression analyses predicting depression were 43%, 46%, and 28%, respectively. Thus, it cannot be concluded that the nonsignificant findings reflect a true absence of a moderator effect by diagnostic group, or if this arose from a lack of power in this study. Second, the BPD+AVH group included significantly more females than the SZ+AVH group. The sex difference between the groups reflects typical presentation rates in clinical settings, as BPD is more frequently diagnosed in female patients (57), whereas psychotic disorders are more frequently diagnosed in male patients (58). However, we cannot rule out that the results of the current study were influenced by the sex difference between the diagnostic groups. Third, negative appraisals of voices and negative voice content were focused upon as determinants of depression and anxiety in youth with AVH, and did not consider other possible predictors, such as childhood trauma (56), experiential avoidance (59), psychological flexibility and nonjudgmental acceptance (21), meta-cognitive beliefs (60), attachment style (19, 61), interpersonal schema (17, 41), and dissociation (62). Fourth, recently, Strauss et al. (63) reported an alternative factor structure for the BAVQ-R, suggesting that malevolence and omnipotence form a combined factor (“persecutory beliefs”), as do items assessing emotional and behavioral response to voices. Future studies investing cognitive, emotional, and behavioral responses to voices in individuals with BPD should consider using these alternative BAVQ-R subscales. Fifth, depression and anxiety were focused upon as outcome variables in the current study. Recent evidence indicates that AVH in BPD is associated with more suicidal plans and attempts (26), and nonsuicidal self-harm (30). Future research is needed to investigate whether negative appraisals of voices and negative voice content are also predictors of these outcome variables. Finally, due to the cross-sectional design of the study, causal conclusions cannot be drawn regarding the relationship between cognitive appraisals of voices and negative voice content on the one hand, and depression and anxiety on the other.

Clinically, the results of the current study indicate that AVH in youth with BPD should not be marginalized with terms such as “pseudo-hallucinations,” “quasi-psychotic,” or “psychotic-like” symptoms (64, 65), as they are associated with negative appraisals of voices and high levels of depression and anxiety. Instead, when youth with BPD disclose hearing voices, clinicians should intervene early through appropriate diagnosis and treatment. However, clinicians might wonder how best to treat AVH in youth with BPD, as there are no clinical guidelines available. For patients with SZ, antipsychotic medication is the treatment of first choice, often in conjunction with psychological interventions (66, 67). However, no randomized-controlled trial (RCT) has tested whether conventional pharmacotherapy for AVH in SZ is applicable to AVH in BPD (68). To address this important question, our group is conducting the first RCT on aripiprazole in youth with BPD and AVH (69). With regard to psychological interventions, the results of the current study indicate that changing appraisals of supremacy of voices, along with negative voice content, could lead to a reduction in depression among youth voice-hearers, including those with BPD. As it is difficult to change the emotional content of voices directly, cognitive behavioral therapy (CBT) traditionally attempts to achieve a reduction in distress by working on the hearer’s beliefs about the meaning of the voices, through methods such as cognitive restructuring, behavioral experiments designed to test alternative explanations, and the development of more adaptive coping strategies (67). In addition, new therapy approaches within the CBT framework have been developed to specifically address voice content (66), such as cognitive therapy for command hallucinations (70), competitive memory training for humiliating voices (71), and compassionate mind training for critical voices (72). However, although CBT and related interventions have been demonstrated to be effective in treating AVH in SZ (66, 67, 73), to the authors’ knowledge, no RCT to date has investigated its efficacy on voice-hearing in BPD. Thus, while accumulating evidence indicates that individuals with BPD and AVH could benefit from CBT-related interventions (28, 29), future studies are needed to investigate their efficacy in this group.

To conclude, youth with BPD and AVH might hold even more negative beliefs about voices, particularly with regard to supremacy of voices, than those with SZ, and these beliefs are closely linked to depression. Appraisals of voices should be assessed in youth with distressing voices regardless of diagnosis, as they provide an important target for interventions.

## Ethics Statement

In accordance with the Declaration of Helsinki, written informed consent was obtained from all participants, and additionally from a parent or guardian for those under 18 years. The study was approved by the Melbourne Health Human Research Ethics Committee (MHREC2016.086).

## Author Contributions

MC, KT, and AC designed the study. MC and JB collected the data. MC, CH, and HJ analyzed the data and wrote the first draft. All authors contributed to and approved the latest version of the manuscript.

## Funding

MC is supported by grants from the Swiss National Science Foundation (P2BSP1_165354), the Bangerter-Rhyner-Foundation, and the Janggen-Pöhn Foundation.

## Conflict of Interest Statement

The authors declare that the research was conducted in the absence of any commercial or financial relationships that could be construed as a potential conflict of interest.
