# Supplementary material for: Preliminary Evidence for the Cognitive Model of Auditory Verbal Hallucinations in Youth With Borderline Personality Disorder
Source: Front Psychiatry. 2019 May 16;10:292. doi: 10.3389/fpsyt.2019.00292 (PMC6531498; doi:10.3389/fpsyt.2019.00292)
Supplement: Supplementary file 1 [file Table_1.docx]

**Supplementary Material**

**Table 1.** Comparison of the Psychotic Symptom Rating Scales – Auditory Hallucinations (PSYRATS-AH) dimensions according

to Woodward et al. (2014).

|  | BPD+AVH  (n=23) | | | SZ+AVH  (n=20) | | | Group differences | | | | |
| --- | --- | --- | --- | --- | --- | --- | --- | --- | --- | --- | --- |
|  | M (SD) | Mnd | MR | M (SD) | Mnd | MR | | *U* | *p* | ES and (95%) CI |  |
| PSYRATS-AH frequency (frequency, duration, disruption) | 6.74 (2.32) | 6.00 | 21.37 | 6.95 (2.11) | 7.50 | 22.73 | | 215.50 | .721 | 0.47 (0.31, 0.64) |  |
| PSYRATS-AH loudness | 2.43 (0.95) | 2.00 | 22.22 | 2.40 (0.99) | 2.00 | 21.75 | | 235.00 | .898 | 0.51 (0.35, 0.67) |  |
| PSYRATS-AH attribution (location, origin of voices) | 4.30 (1.33) | 4.00 | 19.57 | 4.93 (1.66) | 5.00 | 24.80 | | 174.00 | .165 | 0.38 (0.23, 0.55) |  |
| PSYRATS-AH distress (distress, negative content, control) | 16.00 (2.59) | 16.00 | 23.15 | 14.55 (4.37) | 16.00 | 20.68 | | 256.50 | .515 | 0.56 (0.39, 0.71) |  |

*Notes.* AVH, auditory verbal hallucinations; BPD, borderline personality disorder; SZ, schizophrenia spectrum disorder.

**Table 2.** Relationship between voice frequency, loudness, attribution, and distress with depression

and anxiety (n=43).

|  | DASS-21 Depression | | | | DASS-21 Anxiety | | | |
| --- | --- | --- | --- | --- | --- | --- | --- | --- |
|  | r_s_ | *p* | 95% CI | r_s_ | | *p* | 95% CI |  |
| PSYRATS-AH frequency (frequency, duration, disruption) | .27 | .083 | -0.03, 0.53 | .04 | | .780 | -0.26, 0.34 |  |
| PSYRATS-AH loudness | .20 | .205 | -0.11, 0.47 | .23 | | .141 | -0.08, 0.50 |  |
| PSYRATS-AH attribution (location, origin of voices) | -.06 | .707 | -0.08, 0.50 | -.14 | | .379 | -0.42, 0.17 |  |
| PSYRATS-AH distress (distress, negative content, control) | .34* | .025 | 0.05, 0.58 | .26 | | .098 | -0.04, 0.52 |  |

*Notes.* DASS-21, Depression Anxiety Stress Scale; PSYRATS-AH, Psychotic Symptom Rating

Scales Auditory Hallucinations. Significant at: *, *p* < .05.
